# The mosquito knows no borders: Regional challenges for global confrontation in the dengue battle

**DOI:** 10.1371/journal.pntd.0011830

**Published:** 2024-01-04

**Authors:** Joziana Muniz de Paiva Barçante, José Cherem

**Affiliations:** Department of Medicine, Biomedical Research Center (NUPEB), Federal University of Lavras, Lavras, Minas Gerais, Brazil; Huazhong University of Science and Technology Tongji Medical College, CHINA

Dengue fever is a neglected disease with a global impact, and its incidence and geographical reach are rapidly expanding. Global warming marked by higher average temperatures, precipitation, and longer periods of drought could prompt a record number of dengue fever infections worldwide, due to the thermal biology of mosquitoes [1]. Additionally, increased movement of people, urbanization, and pressure on water and sanitation have driven the spread of dengue fever [2,3]. The absence of access to tap water leads to an increased reliance on water-storing containers, which can facilitate the breeding of mosquitoes. Similarly, the lack of sanitation can contribute to the formation of small pools of stagnant water, serving as ideal breeding grounds for *Aedes* [3].

While more prevalent in the tropical and subtropical regions, this viral infection, transmitted by *Aedes aegypti* and *Ae*. *albopictus* mosquitoes, poses a serious public health problem, with outbreaks increasing in number and severity worldwide [4]. In Europe, *Aedes* mosquito vector species are established in about 22 countries, and dengue fever has been reported for over a decade. In addition to the factors mentioned earlier, the increase in mosquito breeding sites has been identified as a determining factor for the rise in the number of cases. As of 2 October 2023, 74 autochthonous/non-travel-associated dengue cases have been reported in Europe from Italy (42), France (31), and Spain (1) [2,5].

Dengue incidence has significantly increased in recent decades, rising from 8.14 cases per 100,000 inhabitants in 2000 to 67.16 cases per 100,000 inhabitants in 2019. Approximately 50 to 100 million infections occur annually in over 100 endemic countries, putting almost half of the world’s population at risk [6].

In the Americas, over 3 million cases of dengue have already been reported by the middle of 2023. This signifies that as of now, 2023 holds the position of the second-highest annual disease incidence since 1980, when the Pan American Health Organization (PAHO) began collecting data on case numbers [7]. As an aggravating factor, dengue does not have a specific treatment, and the medications prescribed are only meant to control fever and pain.

In spite of the efforts made by the PAHO, which recommended in January 2023 that member countries intensify preparedness and response measures to counter potential dengue and other arbovirus outbreaks, with the aim of averting fatalities and complications arising from these illnesses, what has transpired is a distressing surge in the count of cases and fatalities. This circumstance has resulted in the resignation of the Peruvian Minister of Health [8] and an escalation in mortality rates among children in Bolivia [4]. Moreover, Argentina and Chile (particularly in their northern and central regions) have declared a state of public health emergency in response to the presence of *A*. *aegypti* and the epidemiological conditions prevalent in neighboring nations [9]. Countries like Argentina, Bolivia, and Peru are currently grappling with one of the most severe dengue outbreaks on record. Although no autochthonous cases of dengue have yet emerged in Continental Chile, concerns are mounting over the potential for local transmission.

In Brazil, the situation is no different. Reported cases until the 18th epidemiological week (EW 18) of 2023 are 13% higher than in the same period of 2022 and 73% higher compared to the average of the last 5 years. During the same period, 387 deaths were reported. From the EW 21, 1,515,460 probable cases of dengue were reported in Brazil, representing 76% of all probable cases in the Americas Region, followed by Bolivia with 126,182 and Peru with 115,949 [10].

What is surprising is that this increase is not an isolated event. Dengue outbreaks have been recurring in Brazil for several decades. Similar to what was observed in 2023, in 2022, by the 19th EW, 855,910 suspected dengue cases were reported (incidence rate of 401.2 cases per 100,000 inhabitants), representing an increase of 181.74% compared to the same period the previous year when there were 303,727 probable cases (incidence rate of 143.4 cases per 100,000 inhabitants).

Brazil’s public healthcare system, which represents the sole option for over 75% of Brazilians (165 million people), has been facing saturation since the beginning of the pandemic, impacting both primary care and specialized medical services. We have observed a state of resolute inertia, leading to increased morbidity and mortality from dengue and, consequently, from other diseases. The demand for consultations in primary care centers and emergency units during infectious disease outbreaks leads to a worsening backlog in controlling more prevalent conditions such as hypertension, diabetes, and pulmonary diseases. Longer waiting times for managing various comorbidities result in increased decompensation of these patients, leading to a higher demand for urgent care consultations, more diagnostic tests, and hospitalizations, further burdening an already overwhelmed healthcare system and, consequently, reinforcing this negative cycle that ultimately affects the most socioeconomically vulnerable populations. Neglected diseases like dengue push neglected or socioeconomically vulnerable populations into an even greater abyss, either through the direct effects of the infection or its indirect effects, such as reduced access to public health facilities.

The current dengue emergency may indeed be the result of a combination of favorable climatic conditions resulting from global warming and the vector mosquito’s capacity for adaptation, proliferation, and dispersion [11]. However, it is also evident that, in general, public health policies have been more focused on curative approaches rather than strategic preventive actions, especially for diseases considered neglected. This situation has worsened during the pandemic, as efforts were redirected towards combating COVID-19, leading to a further reduction in surveillance in endemic areas. Now, what is observed is the overlap of a new epidemic with a healthcare system that is fatigued and still undergoing restructuring [12].

We must apply the lessons learned from the pandemic. Collective and effective prevention and control measures are essential and need to be intensified, employing a permanent and more efficient epidemiological surveillance system to guide efforts before seasonal or potential outbreaks occur and also to incorporate strategies to quickly identify an outbreak and direct efforts towards its mitigation [13]. Global diseases require global efforts but also an understanding of regional peculiarities. In this unfortunate scenario, it is essential to highlight that the true extent of the disease burden has been underestimated, as limited access to the healthcare system and diagnostic testing is a reality in many underserved regions. To change this situation, objective, integrated, and systematic measures that take into account the local context and reality are necessary. It is crucial to continuously monitor and track health progress, evaluate the application and funding of emerging prevention and control strategies, and inform evidence-based health policies with a focus on prevention.

Above all, it is essential to remember that mosquitoes do not recognize borders; thus, dengue and other vector-borne diseases will continue to expand throughout the region (and the world). As a consequence of climate change and uncoordinated vector control policies, municipalities or countries sharing borders are directly impacted by the inertia of their neighbors. Integrating research on this important neglected disease at every level—municipal, state, federal, and global—is imperative. Fortunately, the dengue scenario brings forth some new points of hope for controlling this and other significant arboviruses. Regarding dengue vaccines, these have prevented the severity of clinical outcomes but have not halted the infection itself [14], which underscores the need for improvements and field application monitoring. Since Ixchiq was approved as the first chikungunya vaccine on November 2023, it is now urgent for the government to enhance health education initiatives to ensure that there is no reduction in community efforts for vector control following the vaccine’s introduction, as this could compromise the control of other arboviruses such as Zika, for which vaccines are not yet available.

Several vector-targeted strategies have shown promise, including the shifting of naturally occurring allele frequencies, genetic modification through CRISPR-Cas9 technology, and the release of *Wolbachia*-infected *A*. *aegypti*. The latter has been successfully implemented in various parts of the world and is one emerging mosquito control approach that might be largely resistant to warming temperatures [15], resulting in a reduction of dengue incidence by 40% to 96%. Particularly with vector control strategies, it is crucial to conduct comprehensive research to understand potential side effects and assess the associated risks. The safety and efficacy of these approaches need to be meticulously considered to mitigate negative impacts on other diseases and the ecosystem as a whole. Despite the apparent success, all approaches must be continually evaluated and monitored across various scenarios and by experts in entomology and ecology be involved in the evaluation and implementation of these strategies. The existence of differing public policies can potentially compromise collective efforts, as previously stated, insects do not adhere to borders.
